# Adenosine Triphosphate Promotes Allergen-Induced Airway Inflammation and Th17 Cell Polarization in Neutrophilic Asthma

**DOI:** 10.1155/2017/5358647

**Published:** 2017-05-25

**Authors:** Fang Zhang, Xin Su, Gang Huang, Xiao-Feng Xin, E-Hong Cao, Yi Shi, Yong Song

**Affiliations:** ^1^Department of Pulmonary Medicine, Jinling Hospital, Nanjing University School of Medicine, Nanjing 210002, China; ^2^Department of Medical Genetics, The Third Military Medical University, Chongqing 400038, China

## Abstract

Adenosine triphosphate (ATP) is a key mediator to alert the immune dysfunction by acting on P2 receptors. Here, we found that allergen challenge caused an increase of ATP secretion in a murine model of neutrophilic asthma, which correlated well with neutrophil counts and interleukin-17 production. When ATP signaling was blocked by intratracheal administration of the ATP receptor antagonist suramin before challenge, neutrophilic airway inflammation, airway hyperresponsiveness, and Th17-type responses were reduced significantly. Also, neutrophilic inflammation was abrogated when airway ATP levels were locally neutralized using apyrase. Furthermore, ATP promoted the Th17 polarization of splenic CD4^+^ T cells from DO11.10 mice in vitro. In addition, ovalbumin (OVA) challenge induced neutrophilic inflammation and Th17 polarization in DO11.10 mice, whereas administration of suramin before challenge alleviated these parameters. Thus, ATP may serve as a marker of neutrophilic asthma, and local blockade of ATP signaling might provide an alternative method to prevent Th17-mediated airway inflammation in neutrophilic asthma.

## 1. Introduction

The prevalence of allergic asthma, one of the most common airway inflammatory diseases, has increased dramatically over the last three decades around the world [1]. Asthma is generally thought to be a chronic immune inflammatory disease characterized by the expression of both Th2 and Th17 cytokines. Both clinical and experimental evidence support that reciprocal regulation between Th2 and Th17 pathways play an important role in the pathogenesis of asthma [2]. However, recent studies show that Th17 cells are involved in the development of neutrophilic asthma, which is a leading phenotype of clinical asthmatic patients, and contribute to severe and fatal asthma exacerbations and steroid therapy resistance [3]. The molecular mechanism responsible for the abnormal Th17 polarization in neutrophilic asthma remains elusive.

Damage-associated molecular patterns (DAMPs), such as adenosine triphosphate (ATP), contribute to the induction of an immune response to allergens by activation and recruitment of various inflammatory cell types [4]. Several studies suggest that close collaboration between ATP and the activation of dendritic cells is needed to initiate a Th2 immune response to allergens [4, 5]. Recent study demonstrated an important role of ATP in the development and maintenance of Th17-associated inflammatory bowel disease [6]. Therefore, it is of great importance to further research the possible role of ATP in the development of Th17-dysregulated immune responses in neutrophilic asthma.

In this study, we evaluated the potential of ATP to induce Th17 polarization and airway inflammation in a mouse model of experimental neutrophilic asthma. First, we found that blockade of ATP signaling with suramin, a broad-spectrum antagonist of ATP receptors, reduced airway hyperresponsiveness, Th17 responses, bronchoalveolar lavage fluid (BALF) neutrophilia, and airway inflammation. Also, neutrophilic inflammation was abrogated when airway ATP levels were locally neutralized using apyrase, an ATP diphosphohydrolase. Interestingly, ATP treatment promoted the polarization of Th17 cells from splenic CD4^+^ T cells of DO11.10 mice in vitro. Finally, we showed that administration of suramin before challenge alleviated ovalbumin- (OVA-) induced neutrophilic inflammation and Th17 polarization in DO11.10 mice. Our results suggest that ATP is an important inflammatory mediator that regulates airway inflammation and Th17 polarization in neutrophilic asthma.

## 2. Methods

### 2.1. Mice

Eight to 10-week-old female C57BL/6 and DO11.10 mice were obtained from the Animal Center of Jinling Hospital. DO11.10 mice are transgenic for an OVA-specific TCR. All mice were bred and maintained under pathogen-free conditions at 20–22°C with 50–60% humidity. Dark/light cycles were 12 h each. All animal experiments conformed to the principles for the care and use of animals approved by the Institutional Animal Care and Use Committee.

### 2.2. Protocols for the Establishment of the Mouse Model and Experimental Interventions

**Figure 1:**
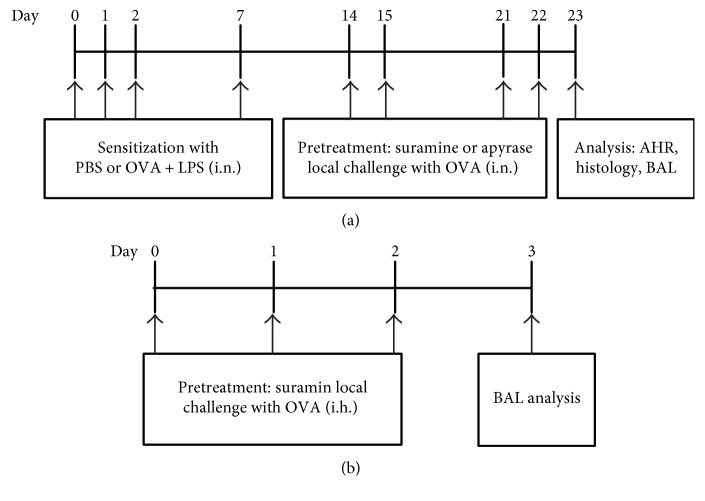
(a) Protocols for the establishment of the OVA and LPS-induced murine model of neutrophilic asthma and experimental interventions. Mice were intranasally sensitized with OVA plus LPS on days 0, 1, 2, and 7 and then challenged with OVA on days 14, 15, 21, and 22. Suramin or apyrase was administered intratracheally at 30 min before each OVA challenge. One day after the final challenge, the mice were sacrificed for analyses. (b) Protocols for establishment of the murine model of neutrophilic asthma and experimental interventions in DO11.10 mice. DO11.10 mice were challenged with an aerosolized solution of OVA on days 0, 1, and 2, and suramin was administered intratracheally at 30 min before each OVA challenge. One day after the final challenge, the mice were sacrificed for analyses.

A murine model of neutrophilic asthma characterized by Th17 cell responses was established as described previously [7]. Briefly, mice received intranasal sensitization with 75 *μ*g ovalbumin (OVA, grade V; Sigma-Aldrich, St Louis, MO, USA) plus 10 *μ*g lipopolysaccharide (LPS, *E. coli* serotype 026:B6; Sigma-Aldrich) on days 0, 1, 2, and 7 and were then challenged by intranasal instillation of 50 *μ*g OVA alone on days 14, 15, 21, and 22. All mice were analyzed at 24 h after the last OVA challenge. OVA reagent was endotoxin tested with limulus amebocyte lysate (ZhanJiang A&C Biological, China) and contained 0.001 EU/mg endotoxin content. Thus, this product is considered to be endotoxin free. The mice were randomly divided into four groups (*n* = 3 ~ 5 mice) as follows: (i) mice sensitized with phosphate-buffered saline (PBS) and challenged with OVA (control group); (ii) mice sensitized with OVA plus LPS and challenged with OVA (asthma group); (iii) mice sensitized with OVA plus LPS and treated with 20 *μ*L PBS at 30 min before the same challenge with OVA (vehicle group); and (iv) mice sensitized with OVA plus LPS and treated with suramin (Sigma-Aldrich) at 30 min before the same challenge with OVA (suramin group). Suramin (200 *μ*M) in 20 *μ*L PBS was administered intratracheally on days 14, 15, 21, and 22 before challenge (Figure 1(a)). In some experiments, mice from the asthma group received an intratracheal (i.t.) injection of 4 U/mL apyrase (Sigma-Aldrich) 30 min before each OVA exposure. Twenty-four hours after the last OVA exposure, bronchoalveolar lavage fluid (BALF) and lung tissue were collected for use later.

In wild-type mice, OVA sensitization and subsequent aerosol challenge generate an eosinophilic inflammation associated with Th2 cytokine expression. By contrast, in DO11.10 mice, the same treatment fails to induce eosinophilia, but instead promotes lung neutrophilia [8]. In this study, an ovalbumin- (OVA-) induced neutrophilic asthma in DO11.10 mice was established as described previously [9]. In brief, mice were challenged with an aerosolized solution of 3% OVA (Sigma-Aldrich, St. Louis, MO) for 10 min from days 0 to 2, and all mice were sacrificed for analysis on day 3. For the blocking ATP-induced Th17 polarization, anaesthetized mice received an intratracheal injection of suramin (200 *μ*M) at 30 min before each OVA challenge.

### 2.3. BALF

To assess differential BALF cell counts, the lungs were lavaged three times with 0.75 mL Ca^2+^- and Mg^2+^-free Hank's balanced salt solution containing 0.1 mM sodium EDTA. The BALF samples were centrifuged at 300*g* for 5 min to obtain a cell pellet and cell-free supernatant that was stored at −80°C until analysis by enzyme-linked immunosorbent assays (ELISAs). For differential cell counts, cell pellets were fixed and stained with Diff-Quick (Siemens HealthCare Diagnostics, Tarrytown, NY, USA) and differentiated morphologically by counting 300 cells/slide. The levels of interleukin IL-17A, IL-4, and interferon-gamma (IFN-*γ*) in the BALF were determined by ELISAs according to the manufacturer's instructions (eBioscience).

### 2.4. Histological Examination

For histopathological assessment, the nonlavaged lobe of the lung tissues were fixed with 10% buffered neutral formalin, embedded in paraffin, cut into 5 *μ*m sections, and stained with hematoxylin and eosin (H&E) using 0.2% hematoxylin for 5 min and 0.35% eosin for 3 min or Masson using 1% hematoxylin for 6 min and ponceau-acid fuchsin staining solution (0.7% ponceaux, 0.3% acid fuchsin) for 1 min. The sections were observed under a microscope at ×200 magnification. Inflammatory changes were graded on a scale of 0 to 5 for perivascular and bronchiolar eosinophilia, epithelial damage, and edema [10]. The collagen areas of similar size bronchioles were analyzed using Image-Pro Plus 4.5 (Media Cybernetics Inc., Rockville, MD, USA) and are presented as positive area/total area of bronchioles [11].

### 2.5. Airway Hyperreactivity (AHR)

AHR was assessed in conscious unrestrained mice by whole-body plethysmography (model: PLY 3211; Buxco Electronics, Troy, NY, USA) as described previously [12]. Briefly, the mice were placed in the plethysmograph chamber and exposed to an aerosolized normal saline solution (baseline readings) and then to cumulative concentrations of *β*-methacholine ranging from 3 to 24 mg/mL. The aerosol was generated using an ultrasonic nebulizer and drawn through the chamber for 2 min. The inlet was then closed, and enhanced pause (Penh) readings were recorded for 3 min and averaged. The values were then calculated as the Penh index.

### 2.6. Measurement of ATP

The ATP concentration in BLAF was measured as described previously [13]. After the samples were collected and centrifuged, the levels of ATP in supernatants were determined by a luciferin-luciferase assay using an ATP assay kit (Thermo-Scientific, Waltham, MA, USA) according to the manufacturer's instructions.

### 2.7. Treatment of Splenic Cells with ATP-*γ*S

Splenic cells were prepared from the spleens of mice as described previously [14]. Splenocytes (1 × 10^5^/well) from DO11.10 mice were stimulated with OVA_323–339_ peptide (50 mmol/L) and/or ATP-*γ*S (100 *μ*M) in a 96-well plate. ATP-*γ*S is a stable ATP derivative and slowly degraded by hydrolytic enzyme. After 5 days of treatment, the culture supernatants were analyzed for IL-17A by ELISAs and the expression of intracellular IL-17 in CD4^+^ T cells was analyzed by flow cytometry. As indicated, graded concentrations of suramin (100, 200, and 400 *μ*M) were added to the cells, and all parameters mentioned above were analyzed.

### 2.8. Flow Cytometric Analysis

To detect IL-17^+^/CD4^+^ T cells among splenic cells, the cells were incubated with 10 *μ*g/mL brefeldin A (eBioscience) for 2 h and then stained for cell surface CD4 using a FITC-conjugated anti-CD4 monoclonal antibody (mAb) (eBioscience) at 4°C for 30 min. After incubation in fixation/permeabilization solution (eBioscience), the cells were stained for intracellular IL-17 using a PE-conjugated anti-IL-17 mAb (eBioscience) for 30 min and then analyzed using a FACSCalibur flow cytometer (BD Biosciences).

To detect IL-17^+^/CD4^+^ T cells in the lung tissue, lung cells were obtained according to a previously reported method [15]. Lung cells (4 × 10^6^/mL) were washed three times in FACS buffer (PBS containing 1% bovine serum albumin and 0.1% sodium azide), incubated with brefeldin A (10 *μ*g/mL) for 2 h, and then stained with surface-specific mAbs (anti-CD3-APC and anti-CD4-FITC; eBioscience) for 30 min at 4°C. For intracellular staining, cells were fixed and permeabilized in fixation/permeabilization solution, intracellularly stained with the PE-conjugated anti-IL-17 mAb for 30 min, and then analyzed using the FACSCalibur flow cytometer.

### 2.9. Statistical Analysis

Data are expressed as the mean ± standard error of mean. Differences between groups were analyzed by the unpaired, two-tailed, parametric Student's *t*-test or analysis of variance using SPSS for windows (version 17.0). *P* values of less than 0.05 were considered to be statistically significant.

## 3. Results

### 3.1. Levels of ATP and P2X7R Expression Are Significantly Increased in Asthmatic Mice

**Figure 2:**
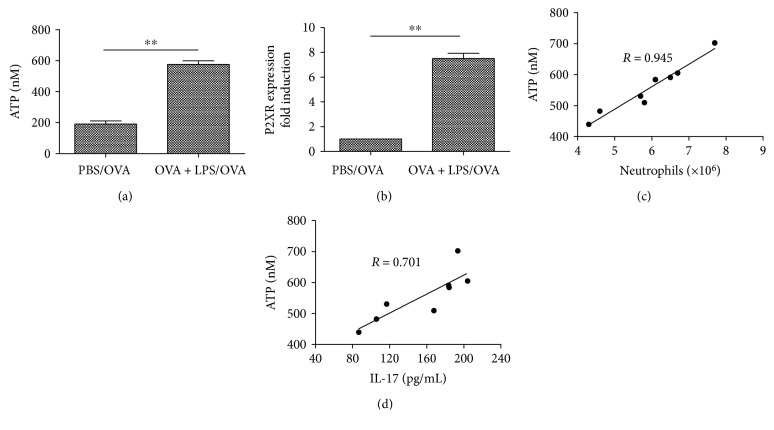
Levels of ATP and P2X7R expression are significantly increased in the mouse model of neutrophilic asthma. (a) ATP levels analyzed by a luciferin-luciferase assay in the BALF. The values represent the means ± SEM (*n* = 6). ^∗∗^*P* < 0.01 compared with the PBS/OVA group. (b) P2X7R expression analyzed by with quantitative PCR in the lung tissue. The values represent the means ± SEM (*n* = 6). ^∗∗^*P* < 0.01 compared with the PBS/OVA group. (c) Positive correlation between the levels of ATP and neutrophils (*R* = 0.945, *P* < 0.01) and (d) a relatively lower correlation between ATP and IL-17 levels (*R* = 0.701, *P* < 0.05) in BALF from OVA plus LPS-sensitized mice. Data are the mean ± SEM; *n* = 8 mice in each group.

Using a mouse model of neutrophilic asthma induced by OVA plus LPS sensitization followed by three sequential daily OVA challenges, we investigated the functional relevance of ATP levels and allergic Th17 responses to the disease. As shown in Figure 2(a), there was a significant increase in ATP levels of BALF from mice sensitized with OVA plus LPS compared with PBS-primed controls. Moreover, ATP levels in OVA plus LPS-sensitized mice, but not in PBS-sensitized mice, correlated well with neutrophil counts (Figure 2(c)) and IL-17 levels (Figure 2(d)) in BALF. These results suggest a strong association of ATP levels with the pathogenesis of experimental neutrophilic asthma.

The purinergic P2 receptors (P2XRs or P2YRs) sense ATP released during cell damage-activation, thus initiate innate proinflammatory inflammation [16]. The key P2 receptor involved in inflammation was identified as P2X7R. Some studies showed a potential role of P2X7R signaling in the initiation of psoriasis pathogenesis, islet allograft rejection, and rheumatoid arthritis (RA) [17–19]. Moreover, the activation of P2X7R resulted into Th17 differentiation and the subsequent induction of Th17-biased immunity. To better define the role of P2X7R signaling in asthmatic airway inflammation, we analyzed the expression of the P2X7R subtype in the lung tissue with Quantitative PCR. The results demonstrated that acute allergic airway inflammation was associated with a strong upregulation of the P2X7R subtype in lung tissue (Figure 2(b)).

### 3.2. Local Treatment with Suramin Reduces Neutrophilic Airway Inflammation and Remodeling

**Figure 3:**
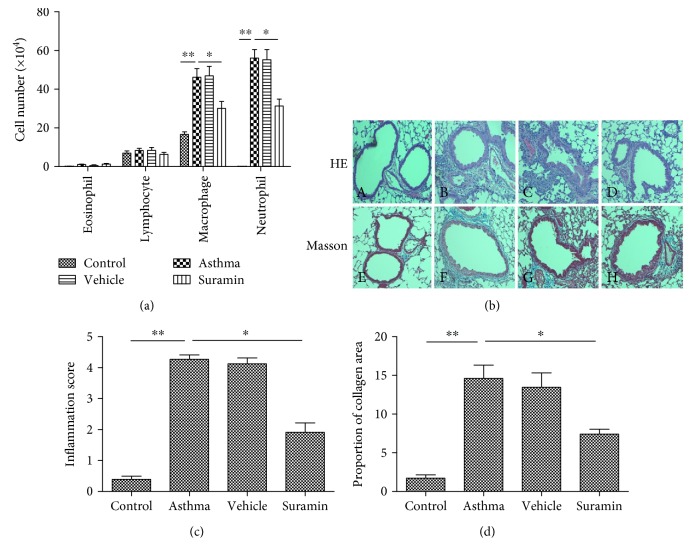
Local administration of suramin inhibits neutrophilic airway inflammation and remodeling. (a) Suramin inhibited the infiltration of neutrophils and macrophages into BALF. BALF was collected at 24 h after the last OVA challenge. Differential cells were counted in the BALF of mice in control, asthma, vehicle-treated, and suramin-treated groups. The values represent the means ± SEM (*n* = 4 ~ 5). ^∗^*P* < 0.05 and ^∗∗^*P* < 0.01 compared with the asthma group. (b) Representative H&E and Masson staining of lung tissues. H&E-stained lung tissues (upper panel) from mice in control—A, asthma—B, vehicle-treated—C, and suramin-treated groups—D. Masson-stained lung tissues (lower panel) from mice in control—E, asthma—F, vehicle-treated—G, and suramin-treated groups—H. (c) Inflammation scores were decreased in suramin-treated mice. The values represent the means ± SEM (*n* = 4 ~ 5). ^∗^*P* < 0.05 and ^∗∗^*P* < 0.01 compared with the asthma group. (d) The proportion of collagen area was decreased in suramin-treated mice. The values represent the means ± SEM (*n* = 4 ~ 5). ^∗^*P* < 0.05 and ^∗∗^*P* < 0.01 compared with the asthma group.

Because the increase in ATP levels and P2X7R expression of asthmatic mice was closely related to neutrophil infiltration and IL-17 production, we next determined whether blocking ATP would decrease neutrophilic inflammation and Th17 cytokine production in asthmatic mice. To evaluate the effects of ATP on allergen-primed airway inflammation, we intratracheally administrated suramin, an antagonist of ATP receptors, before OVA challenge. As expected, there were substantial increases in the neutrophils and macrophages of BALF in OVA plus LPS-sensitized mice upon subsequent OVA challenge, but small amount of eosinophils, which is a characteristic of neutrophilic bronchial asthma. However, treatment with suramin before OVA challenge led to significant decreases in the numbers of neutrophils and macrophages in BALF compared with vehicle-treated mice (Figure 3(a)).

To further characterize the effects of suramin on OVA-induced airway inflammation and remodeling, we also compared lung histologies among the various groups of mice. As shown in H&E-stained sections (Figure 3(b), upper panel), OVA plus LPS-sensitized mice showed increased numbers of inflammatory cells in the peribronchial and perivascular regions, which were rarely detected in sham-sensitized mice. In contrast, suramin administration significantly alleviated airway inflammation because little peribronchiolar and perivascular infiltrates were found in the airways. Inflammation scores were used to assess the infiltration extent of inflammatory cells in the lungs (Figure 3(c)). The scores of the suramin-treated mice were lower than those of the vehicle-treated mice (*P* < 0.05). In addition, Masson's staining was used to assess collagen deposition and airway remodeling. After Masson's staining of the lung tissue, smooth muscle and red blood cells were red, and collagen fibers were blue (Figure 3(b), lower panel). The bronchus morphometry measurement results showed that OVA-induced collagen deposition in the lungs was markedly reduced by suramin treatment (Figure 3(d)). Collectively, local administration of suramin in the mouse model of neutrophilic asthma substantially lessened the severity of neutrophilic airway inflammation and remodeling.

### 3.3. Neutralizing Airway ATP with Apyrase Reduces Airway Inflammation

**Figure 4:**
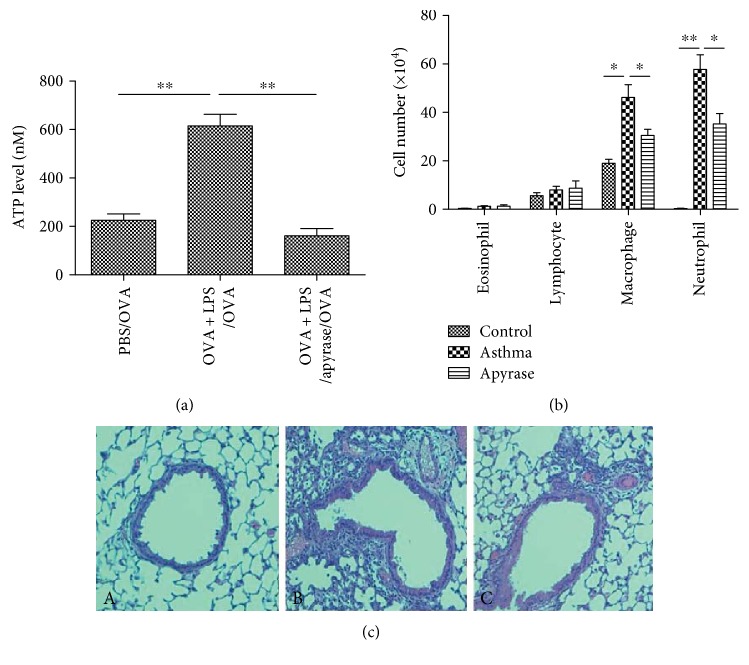
Local administration of suramin inhibits neutrophilic airway inflammation. (a) Instillation of apyrase-neutralized airway ATP. Apyrase was intratracheally administered into mice 30 min before allergen challenge, and ATP levels in the BALF were analyzed by a luciferin-luciferase assay. The values represent the means ± SEM (*n* = 4 ~ 5). ^∗^*P* < 0.05 and ^∗∗^*P* < 0.01 compared with the asthma group. (b) Apyrase inhibited the infiltration of neutrophils and macrophages into BALF. BALF was collected at 24 h after the last OVA challenge. Differential cells were counted in the BALF of mice in control, asthma, and apyrase-treated groups. The values represent the means ± SEM (*n* = 4 ~ 5). ^∗^*P* < 0.05 and ^∗∗^*P* < 0.01 compared with the asthma group. (c) Representative H&E staining of lung tissues. H&E-stained lung tissues from mice in control—A, asthma—B, and apyrase-treated groups—C. The peribronchial and perivascular inflammations were decreased in apyrase-treated mice.

We next neutralized airway ATP by administering the ATP-hydrolyzing enzyme apyrase into intratracheal of mice 30 min before allergen challenge. Instillation of apyrase greatly lowered the concentration of ATP in BALF (Figure 4(a)), and this was accompanied by a decrease in neutrophils and macrophages (Figure 4(b)), as well as peribronchial and perivascular inflammations (Figure 4(c)). Because suramin may block other receptors besides the P2R family (e.g., sphingosine-1-phosphate receptor-3) [20], we used the ATP-hydrolyzing enzyme apyrase, which also reduced the airway inflammation of asthma, illustrating ATP has potential to promote the airway inflammation.

### 3.4. Suramin Inhibits the Development of AHR

**Figure 5:**
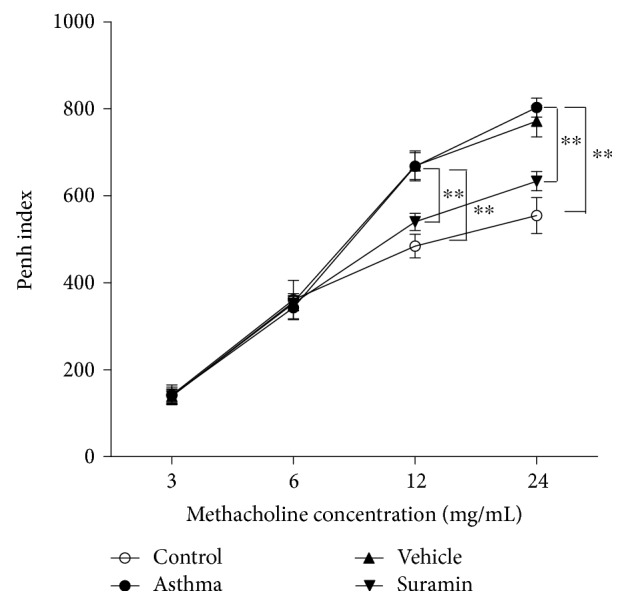
Local administration of suramin decreases AHR. The Penh index represents the OVA-induced AHR to methacholine, which was measured in mice from control, asthma, vehicle-treated, and suramin-treated groups using a body plethysmograph. Values represent the means ± SEM (*n* = 3 ~ 5). ^∗∗^*P* < 0.01 compared with the asthma group.

To assess the effect of suramin on AHR, we evaluated the relative increase of Penh in response to methacholine inhalation. After OVA challenge, AHR was increased in OVA plus LPS-sensitized mice upon subsequent OVA challenge. Administration of suramin before the challenge phase significantly inhibited the development of AHR (Figure 5).

### 3.5. Suramin Suppresses Local Th17 Responses In Vivo

**Figure 6:**
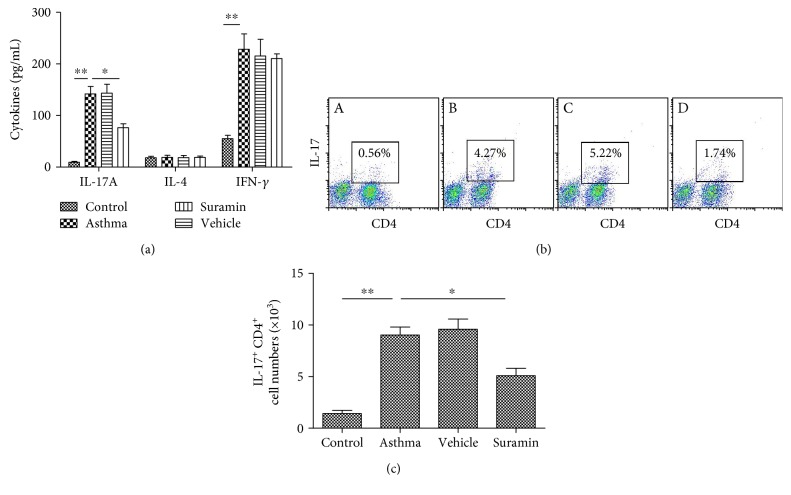
Local administration of suramin suppresses the Th17 response. (a) Suramin decreased Th17 cytokine production after OVA challenge. BALFs were collected at 24 h after the last OVA challenge. The levels of IL-17A, IL-4, and IFN-*γ* in the BALF of mice from control, asthma, vehicle-treated, and suramin-treated groups were assessed by ELISAs. The values represent the means ± SEM (*n* = 3 ~ 5). ^∗^*P* < 0.05 and ^∗∗^*P* < 0.01 compared with the asthma group. (b) Suramin decreased Th17 cell counts in the lung. The percentages of Th17 cells among CD3^+^-gated lung cells from mice in control—A, asthma—B, vehicle-treated—C, and suramine-treated groups—D were analyzed by flow cytometry. Representative results are shown. (c) Histogram showing the absolute counts of Th17 cells among the lung cells of mice from the various groups. Values represent the means ± SEM. ^∗^*P* < 0.05 and ^∗∗^*P* < 0.01 compared with the asthma group.

To analyze the immune response in the airway, we measured the cytokine levels in BALF. As shown in Figure 6(a), local administration of suramin resulted in remarkable reductions in the levels of IL-17A (Th17-associated cytokines). In contrast, there were no significant differences in the levels of IL-4 (Th2-associated cytokine) and IFN-*γ* (Th1-associated cytokine) compared with mice that received PBS.

### 3.6. Suramin Decreases Th17 Cell Proportion in the Lung Tissue

Because administration of suramin decreased the IL-17A level in BALF, we investigated the possibility that such inhibition was mediated through a decrease in the proportion of Th17 cells in the lung. As shown in Figures 6(b) and 6(c), OVA plus LPS-sensitized mice exhibited a dramatic increase of Th17 cell proportion in the lung tissue, whereas administration of suramin significantly inhibited the proportion of Th17 cells in the lung tissue. These results suggest that local administration of suramin suppresses Th17 skewing in the mouse model of neutrophilic asthma.

### 3.7. ATP Stimulates Allergen-Induced Th17 Responses In Vitro

**Figure 7:**
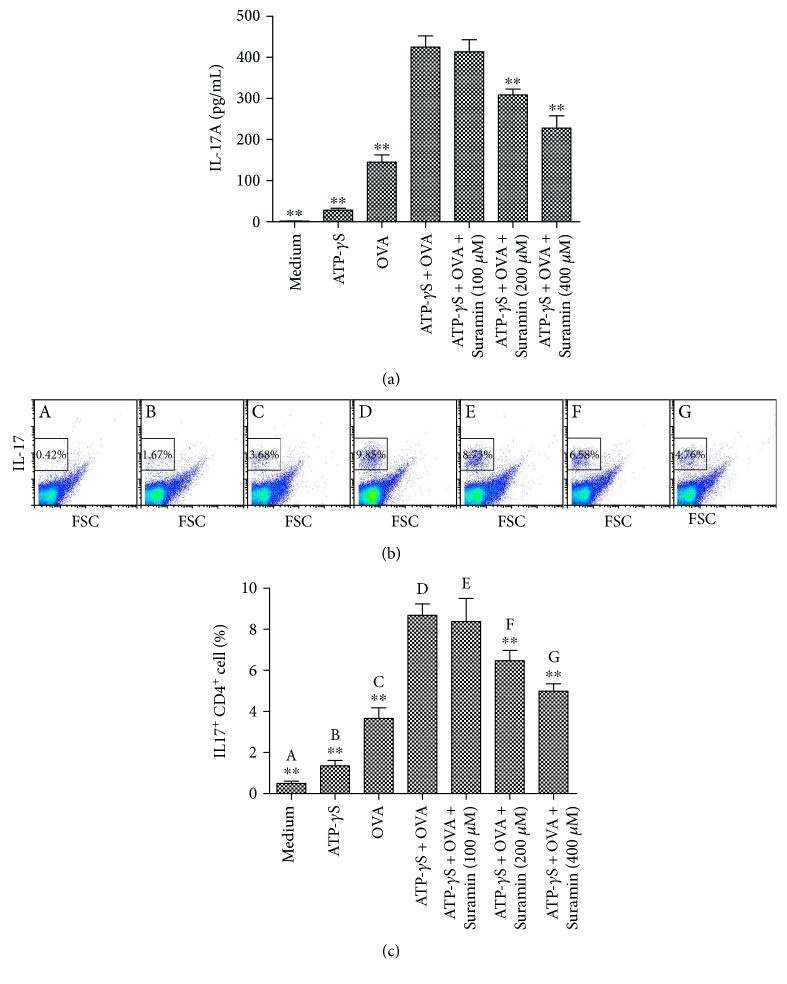
Suramin inhibits the Th17 response of ATP-*γ*S and OVA_323–339_ peptide-stimulated splenocytes from DO11.10 mice in vitro. (a) Suramin inhibited production of IL-17A in ATP-*γ*S and OVA_323–339_ peptide-stimulated splenocytes. The splenocytes from DO11.10 mice were cultured in medium only, stimulated with OVA_323–339_ peptide (50 mmol/L) or (and) ATP-*γ*S (100 *μ*M), or treated with graded concentrations of suramin (100, 200, and 400 *μ*M) for 5 days. IL-17A levels in the supernatants were measured using ELISAs. The values represent the means ± SEM (*n* = 5). ^∗∗^*P* < 0.01 compared with the ATP-*γ*S and OVA_323–339_ peptide-stimulated splenocyte group. (b, c) Suramin inhibited Th17 polarization induced by ATP-*γ*S and OVA_323–339_ peptide-stimulated splenocytes. The percentages of IL-17^+^ CD4^+^ T cells were analyzed by flow cytometry (b). (c) Histogram showing the percentages of IL-17^+^ CD4^+^ T cells after 5 days of treatment. Values represent the means ± SEM (*n* = 5). ^∗∗^*P* < 0.01 compared with the ATP-γS and OVA_323–339_ peptide-stimulated splenocyte group.

To examine the contribution of ATP to Th17 development in vitro, Th17 polarization was analyzed by treatment of splenocytes from DO11.10 mice with OVA_323–339_ peptide in the absence or presence of ATP-*γ*S. We found that ATP-*γ*S combined with OVA_323–339_ peptide significantly enhanced IL-17A levels (Figure 7(a)) in the culture supernatants and increased the percentage of IL-17^+^ CD4^+^ T cells (Figures 7(b) and 7(c)). In contrast, high concentrations of suramin (200 or 400 *μ*M) significantly downregulated the expression of IL-17 induced by ATP-*γ*S combined with OVA_323–339_ peptide, suggesting that suramin suppressed the ATP-stimulated and specific allergen-induced Th17 response.

### 3.8. ATP Induces Th17 Priming In Vivo

**Figure 8:**
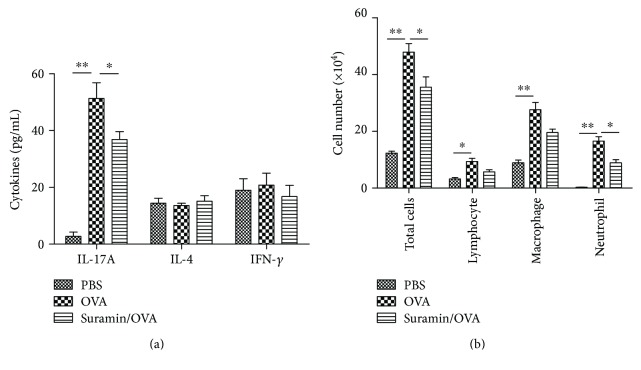
Suramin impedes the potential of ATP-induced Th17 responses in vivo. DO11.10 mice were challenged with an aerosolized solution of PBS or OVA from days 0 to 2, and suramin was intratracheally administered at 30 min before each OVA challenge. All mice were sacrificed for analysis on day 3. (a) Total and differential cells counts and (b) cytokine profiles were analyzed in the BALF. Values represent the means ± SEM (*n* = 5). ^∗^*P* < 0.05 and ^∗∗^*P* < 0.01 compared with the OVA group.

Some studies have shown a potential induction of Th17 response in the neutrophil-promoting inflammation, such as neutrophilic asthma [21, 22]. To evaluate the role of ATP in Th17 polarization in vivo, we measured the ATP levels of BALF in DO11.10 mice by OVA-induced neutrophilic asthma and administrated suramin before the challenge to evaluate its effects on the Th17 response and airway inflammation. The results showed a remarkable increase of ATP level in the airway of asthmatic mice, but local administration of suramin had no significant impact on ATP level (data not shown). Further, OVA challenge induces Th17/Th1 priming as shown by increases in IL-17A, and IFN-*γ* levels in the BALF (Figure 8(a)) and significant neutrophilic inflammation were characterized by infiltration of neutrophils into the BALF (Figure 8(b)) in DO11.10 mice. However, administration of suramin markedly decreased the IL-17A levels and the infiltration of neutrophils in BALF (Figures 8(a) and 8(b)). These data suggest that suramin inhibits ATP-induced neutrophilic inflammation and Th17 skewing in asthmatic mice.

## 4. Discussion

Neutrophilic asthma has been recently described as a different inflammatory subtype of asthma, which is characterized by neutrophilic rather than eosinophilic airway inflammation and airway hyperresponsiveness [23, 24]. Increased Th17 immune responses correlate with the pathogenesis of neutrophilic asthma [15, 25]. However, the Th17 function in neutrophilic asthma as a driving mechanism of neutrophilic inflammation is not yet fully understood and requires further exploration. ATP has been described as orchestrating allergic inflammation and the immune response in allergic disease based on studies in animal models [26]. In fact, ATP is a DAMP that triggers an immediate innate immune response through activation of innate immune cells, including neutrophils, monocytes and macrophages, and dendritic cells [27, 28]. ATP signaling is strongly associated with various inflammatory and autoimmune disorders such as inflammatory bowel disease and rheumatoid arthritis [29, 30]. Therefore, we postulated that ATP contributes to the regulation of immune responses in neutrophilic asthma. Idzko et al. have reported that the level of ATP in BALF is positively correlated with markers of airway inflammation in eosinophilic asthma, and all the cardinal features of asthma, including airway inflammation, Th2 cytokine production, and bronchial hyperreactivity, were abrogated when lung ATP levels were locally neutralized using apyrase or when mice were treated with broad-spectrum P2 receptor antagonists. The effects of ATP were due to the recruitment and activation of lung myeloid dendritic cells that induced Th2 responses in the mediastinal nodes [4]. Our study aims to define the role of P2 receptor during neurotrophic asthma and to investigate the potential mechanism of ATP-induced Th17 polarization by using different animal models.

Our data indicate that ATP is involved in neutrophilic asthma. First, ATP levels were increased in BALF from the mouse model of neutrophilic asthmatic. The elevation in ATP levels was highly correlated with elevated IL-17 levels and neutrophil numbers, a cardinal feature of neutrophilic asthma. One study has demonstrated that activated neutrophils infiltrating into inflamed lung tissue are the major source of increased ATP levels [31]. Furthermore, our study showed that administration of suramin inhibited airway neutrophilic inflammation, airway remodeling, and AHR, while neutralizing airway ATP with apyrase also reduces airway inflammation, suggesting that ATP signaling is a key regulator in the immune cascade leading to subsequent Th17 polarization as well as neutrophilic airway inflammation. The Th17 response has been shown to be a reliable biomarker for the diagnosis and prognosis of severe asthma including neutrophilic asthma [22, 32]. Our study indicates that extracellular ATP plays important roles in the development of airway inflammation and Th17 polarization in neutrophilic asthma.

We also investigated whether ATP stimulated Th17 polarization in vitro. Using splenocytes from DO11.10 mice and OVA coculture system, we found that ATP induced IL-17 expression in the culture supernatant and the presence of Th17 cells in a dose-dependent manner, while addition of a P2R-blocking antibody attenuated the stimulatory effect of ATP on Th17 polarization of CD4^+^ T cells. These results indicate that ATP promotes Th17 polarization through purinergic receptors. To determine the effects of ATP-induced Th17 responses in vivo, we performed experiments in which suramin was administrated to the airways of OVA-induced neutrophilic asthma in DO11.10 mice before the challenge phase. As expected, OVA challenge triggered strong neutrophilic airway inflammation that was accompanied by extensive production of Th17 cytokines such as IL-17. However, pretreatment with suramin resulted in significant reductions of neutrophil infiltration as well as IL-17 levels. These data further highlight the putative role of ATP signaling in allergic disease and demonstrate the potential of blocking ATP signaling to control Th17 responses and neutrophilic airway inflammation.

Though we found that ATP stimulated Th17 polarization, further research is necessary to delineate the underlying mechanism. Other study has showed that ATP can induce APC maintaining a distinct activation state favoring IL-23-dependent Th17-type response [33]. We assume that ATP has direct effects on APC and indirect effects on differentiation of Th17. Some work is being processed to determine whether ATP signaling facilitates the development of the APC-derived cytokines that favors a Th17 phenotype. Moreover, suramin is mostly a nonselective inhibitor and has several effects unrelated to its ability to block purinergic receptors [20], further experiments with a more specific P2 receptor blocker, such as PPADS, should be taken into consideration.

In conclusion, our findings demonstrate that ATP has a crucial role in promoting Th17-mediated cardinal features of neutrophilic asthma. Blocking ATP in neutrophilic asthma may be an effective therapeutic strategy to reduce inflammation and prevent neutrophilic asthma.
